# Associations between declines in uneven terrain walking speed and visuospatial working memory in older adults

**DOI:** 10.3389/fnagi.2025.1644741

**Published:** 2026-02-02

**Authors:** Jungyun Hwang, Chang Liu, Steven P. Winesett, Tyler Fettow, Valay A. Shah, Sudeshna A. Chatterjee, Todd M. Manini, Chris J. Hass, Rachael D. Seidler, Daniel P. Ferris, Arkaprava Roy, Patricia A. Reuter-Lorenz, David J. Clark

**Affiliations:** 1 Department of Neurology, University of Florida, Gainesville, FL, United States; 2 Department of Kinesiology and Nutrition, University of Illinois Chicago, Chicago, IL, United States; 3 Department of Biomedical Engineering, University of Florida, Gainesville, FL, United States; 4 McKnight Brain Institute, University of Florida, Gainesville, FL, United States; 5 Brain Rehabilitation Research Center, Malcom Randall VA Medical Center, Gainesville, FL, United States; 6 NASA Langley Research Center, Hampton, VA, United States; 7 Department of Applied Physiology and Kinesiology, University of Florida, Gainesville, FL, United States; 8 Department of Physical Therapy and Rehabilitation Sciences, Drexel University, Philadelphia, PA, United States; 9 Department of Health Outcomes and Biomedical Informatics, University of Florida, Gainesville, FL, United States; 10 Department of Biostatistics, University of Florida, Gainesville, FL, United States; 11 Department of Psychology, University of Michigan, Ann Arbor, MI, United States

**Keywords:** aging, brain, n-back, prefrontal cortical activity, uneven terrain, walking, working memory

## Abstract

**Introduction:**

Mobility and cognitive functions often decline concurrently in older adults, and may be particularly detrimental to walking in complex environments such as uneven terrain. Walking on uneven terrain particularly relies on visuospatial working memory to continuously adjust gait patterns; however, this relationship remains understudied. The objectives of this study are to examine group differences in uneven terrain walking speed and visuospatial n-back task performance across varying task demands among younger adults, and among older adults with high and low physical function and to assess whether uneven terrain-induced reductions in walking speed are associated with declines in n-back performance, particularly among low-functioning older adults. As an exploratory aim, we also examined age-related differences in brain activity during n-back performance to provide additional context for interpreting neural responses across tasks.

**Methods:**

The analysis included 24 younger adults (aged 22.8 ± 3.3) and 44 older adults (aged 74.0 ± 5.6). Using the Short Physical Performance Battery (SPPB), older adults were categorized into high physical function (*n* = 29) and low physical function (*n* = 15), with scores below 10 indicating a lower level of physical function. Uneven terrain walking speed was measured as participants traversed four novel custom-made overground surfaces: flat, low, medium, and high terrain unevenness. Visuospatial walking memory was assessed on a spatial n-back task that included four n-back levels, ranging from 0-back to 3-back. Prefrontal cortical activity was measured using functional near-infrared spectroscopy (fNIRS) while participants performed the n-back task.

**Results:**

Compared to younger adults, older adults exhibited poorer working memory performance and slower uneven terrain walking speeds, with both effects being particularly pronounced in low functioning older adults. Slower walking speeds as terrain became more uneven were associated with poorer n-back performance as n-back level increased, with a larger effect size observed in the low physical functioning older adults. fNIRS results revealed comparable levels of prefrontal cortical activity between younger and older groups during the n-back tasks. Prefrontal cortical activity did not increase with higher task demands (i.e., increasing n-back levels) in any of the groups.

**Conclusion:**

These findings support a relationship between declines in uneven terrain mobility and n-back cognitive function in older adults; however, this relationship was not observed in younger adults. Further research is needed to understand the shared neural mechanisms underlying age-related declines in mobility and cognitive function.

## 1 Introduction

Currently, more than one third of individuals aged 65 or older live with mobility limitations, such as difficulties with walking or climbing stairs (Maresova et al., 2023). Preserving mobility function is important to enable older adults to perform daily activities and maintain their functional independence (Billot et al., 2020). Common age-related mobility limitations, including slower gait speed and balance deficits, are associated with reduced movement quality and a heightened risk of all-cause mortality (Abellan van Kan et al., 2009; Studenski et al., 2011). Emerging evidence indicates that declines in mobility function and cognitive function are closely linked in older adults (Kueper et al., 2017). This relationship may differ depending on an individual's level of physical functioning; however, it remains underexplored, particularly in comparisons between high- and low-physically functioning older adults across varying levels of mobility and cognitive task difficulty. Stratifying older adults by physical function allows for potential identification of differing motor or cognitive contributions to functional outcomes. This approach enables clinically relevant insights, such as selecting the components of interventions to enhance walking function.

Declines in gait speed, a critical aspect of mobility function, have emerged as a significant factor associated with mild cognitive impairment and dementia in older adults (Verghese et al., 2007). Several systematic reviews and meta-analyses consistently highlight a positive association between gait speed and cognitive function in older adults, as demonstrated by both cross-sectional and longitudinal studies (Demnitz et al., 2016; Peel et al., 2019; Knapstad et al., 2023). These studies commonly assess self-paced gait speed, typically on flat surfaces, alongside cognitive measures such as global cognition, executive function, and processing speed. While the overall findings suggest a modest positive relationship between gait speed and cognitive function, some studies report a weak or non-significant association between gait speed and global cognition (de Bruin and Schmidt, 2010; Bruce-Keller et al., 2012; Berryman et al., 2013; Lord et al., 2014), executive function (Hausdorff et al., 2005; van Iersel et al., 2008; de Bruin and Schmidt, 2010), or memory (Hausdorff et al., 2005; Holtzer et al., 2006; van Iersel et al., 2008). These discrepancies may stem, in part, from variations in the methods used to assess walking or cognitive performance. We previously proposed that walking in complex environments, such as uneven terrain with varying heights, textures, and surface compliance, provides a more sensitive assessment by imposing greater cognitive and mobility demands (Clark et al., 2019). Uneven surfaces disrupt sensory feedback and require careful, step-by-step adjustments in foot placement, step length, and joint positioning, thereby increasing both cognitive and postural demands (Downey et al., 2022; Shah et al., 2025). Walking on uneven terrain impedes forward movement, challenges mediolateral stability, increases step variability, and alters preferred gait speed (Voloshina et al., 2013; Downey et al., 2022; Darici and Kuo, 2023). These mobility demands elevate cognitive load, as reflected in heightened brain activity, as individuals must continuously adapt their gait and maintain balance, particularly in older adults or those with compromised mobility (Hwang et al., 2024; Liu et al., 2024). Recent studies have shown that preferred walking speed decreases as terrain unevenness increases, with older adults exhibiting greater reductions compared to younger adults (Downey et al., 2022; Hwang et al., 2024; Liu et al., 2024; Shah et al., 2025). This terrain-induced decline in walking speed is especially pronounced in individuals with mobility limitations.

Visuospatial working memory is essential for the cognitive control of walking, particularly in complex environments such as uneven terrain (Yogev-Seligmann et al., 2008; Kao and Pierro, 2022), where increased caution and precise foot placement are required to maintain balance when stepping onto surfaces of varying height (Downey et al., 2022). It enables moment-to-moment processing and updating of the shape, position, and height of the terrain, allowing individuals to monitor surface changes and adapt foot placement in real time to maintain stability during walking on uneven ground (Menant et al., 2014; Hawkins et al., 2017). This critical role in navigation underscores the importance of assessing visuospatial working memory to examine age-related differences in the cognitive control of walking. The n-back task, a widely used measure of visuospatial working memory, systematically increases cognitive demand to evaluate working memory performance (Kirchner, 1958). In this task, participants determine whether the spatial location of a stimulus is in a specific location (0-back) or matches the location from one, two, or three trials back (1-back, 2-back, or 3-back). Higher n-back levels increase task difficulty by imposing greater demands on working memory. Performance on the 0-back task reflects basic stimulus localization and response execution, providing a baseline for assessing the continual updating, retention, and comparison of spatial locations in working memory required with 1-back or higher conditions (Kane et al., 2007). Working memory relies heavily on the integrity of the prefrontal cortex (Owen et al., 2005; Nissim et al., 2016), which is particularly vulnerable to age-related decline throughout the adult lifespan, even in healthy individuals (Raz and Rodrigue, 2006). Structural and functional changes in the prefrontal cortex associated with aging (Grady, 2012) can also compromise locomotor control during complex walking tasks (Seidler et al., 2010; Clark, 2015).

Assessing brain activity across varying task difficulty is a validated and widely used approach for characterizing age-related differences in neural control during cognitive and mobility tasks, revealing both compensatory and capacity-limited neural responses in older adults (Cabeza et al., 2018; Clark et al., 2019). Neuroimaging studies shows the fronto-parietal network, especially the prefrontal cortex, plays a central role in n-back working memory performance (Nyberg et al., 2009; Lamichhane et al., 2020) and also supports executive control of walking (Seidler et al., 2010). Because the prefrontal cortex undergoes age-related neuropathological changes that weaken these shared executive control networks, declines in cognitive function and mobility function often emerge together (Seidler et al., 2010). Brain activity in this network intensifies with increasing task difficulty (e.g., higher *n*-back levels), reflecting the increasing cognitive demand (Leon-Dominguez et al., 2015; Yeung and Han, 2023). However, other neuroimaging studies (Mattay et al., 2006; Zhu et al., 2024) have reported no increase or even a decrease in prefrontal cortical activity at higher task difficulty levels, possibly reflecting reduced task engagement, poorer performance, or diminished cognitive effort. Cognitive aging literature (Reuter-Lorenz and Cappell, 2008) generally suggests that older adults recruit additional cognitive resources at lower task loads compared to younger adults during the same task. However, as task difficulty increases, older adults reach a resource ceiling earlier, indicating age-related limitations in neural resource availability and efficiency. This over-activation may be compensatory, enhancing performance when effective, or dysfunctional when associated with poorer outcomes (Fettrow et al., 2021). Compensatory upregulation refers to increased or more widespread brain activity in older adults either within task-relevant regions or through recruitment of additional areas in response to short-term increases in cognitive demands (Cabeza et al., 2002, 2018) and mobility demands (Hwang et al., 2024; Liu et al., 2025). In this study, we measured prefrontal cortical activity during n-back performance under increasing task difficulty, but not during uneven terrain walking speed performance, as the walking period was too brief to obtain reliable cortical activity estimates.

We aimed to compare load-dependent changes in uneven terrain walking speed and n-back performance across three participant groups: younger adults, older adults with high physical function, and older adults with low physical function. We also aimed to examine the relationship between changes in uneven terrain walking speed and n-back performance within each group. Physical function status was assessed using the Short Physical Performance Battery (SPPB; Guralnik et al., 1994), which evaluates balance, gait speed, and lower-extremity strength, with scores below 10 indicating greater mobility limitations. Additionally, we investigated the connection between n-back task performance and prefrontal cortical activity, using functional near-infrared spectroscopy (fNIRS). Our main hypotheses were: (a) both terrain walking speed and n-back performance would decrease as task difficulty increased, with these effects being more pronounced in older adults with low physical function; (b) slower terrain walking speed and poorer n-back performance would be significantly associated, particularly in the low physical function group. Our secondary hypothesis was that (c) prefrontal cortical activity would increase across all participant groups as n-back task difficulty escalated, with the low physical function group reaching a limit in prefrontal recruitment earlier (i.e., at lower task loads) compared to younger adults.

## 2 Materials and methods

### 2.1 Study design

This study is a part of the larger Mind in Motion study (Clark et al., 2019), which focuses on understanding the neural control of mobility function in older adults. For the data set included here, participants attended two separate visits scheduled within 30 days or less of each other. During the first visit, participants underwent anthropometric measurements, cognitive screening, and mobility function assessments. They also completed a terrain walking speed task, which included four levels of difficulty: flat, low, medium, and high terrain unevenness. In the second visit, participants performed the n-back task, which had four levels of difficulty: 0-back, 1-back, 2-back, and 3-back. Additionally, fNIRS was used to assess changes in brain activity during the n-back task. Further details are provided in the following sections.

### 2.2 Participant inclusion and exclusion criteria

Data from 68 participants were analyzed: 24 healthy younger adults (13 females, 11 males; mean age ± SD = 22.8 ± 3.3 years) and 44 community-dwelling older adults (27 females, 17 males; mean age ± SD = 74.0 ± 5.6 years). Briefly, inclusion criteria comprised age ranges of 20–40 years for younger adults and ≥65 years for older adults. Exclusion criteria involved the presence of mild cognitive impairment [i.e., Montreal Cognitive Assessment (MoCA) score < 26; Nasreddine et al., 2005], walking disability (i.e. 400-m walk test in ≥15 min without assistance; Vestergaard et al., 2009), severe obesity [body mass index (BMI) ≥35], or the presence of serious or unstable medical conditions or historical health issues that would prevent the participant from fully engaging in the cognitive and walking assessments. In the 400-m walk, a 15-min cut-off at usual gait speed has been recommended as an objective measure to screen for and identify mobility limitations (Rolland et al., 2004). Full inclusion and exclusion criteria have been described previously (Clark et al., 2019). All participants provided written consent, and the protocol followed ethical guidelines and was approved by the Institutional Review Board at the University of Florida.

### 2.3 Participant group categorization

Older adults were categorized into their respective physical function groups based on their SPPB scores (Pavasini et al., 2016), with SPPB scores ≥10 indicating high physical function (*n* = 29) and scores < 10 indicating low physical function (*n* = 15). The SPPB test is an assessment of global physical function, which includes balance, strength, and gait measurements (Guralnik et al., 1994). This test involves a 4-meter usual pace walk, time to complete five unassisted chair stands, and three standing balance tasks with feet together and in semi- and full-tandem foot positions.

### 2.4 Assessment of walking speed during uneven terrain walking

**Figure 1:**
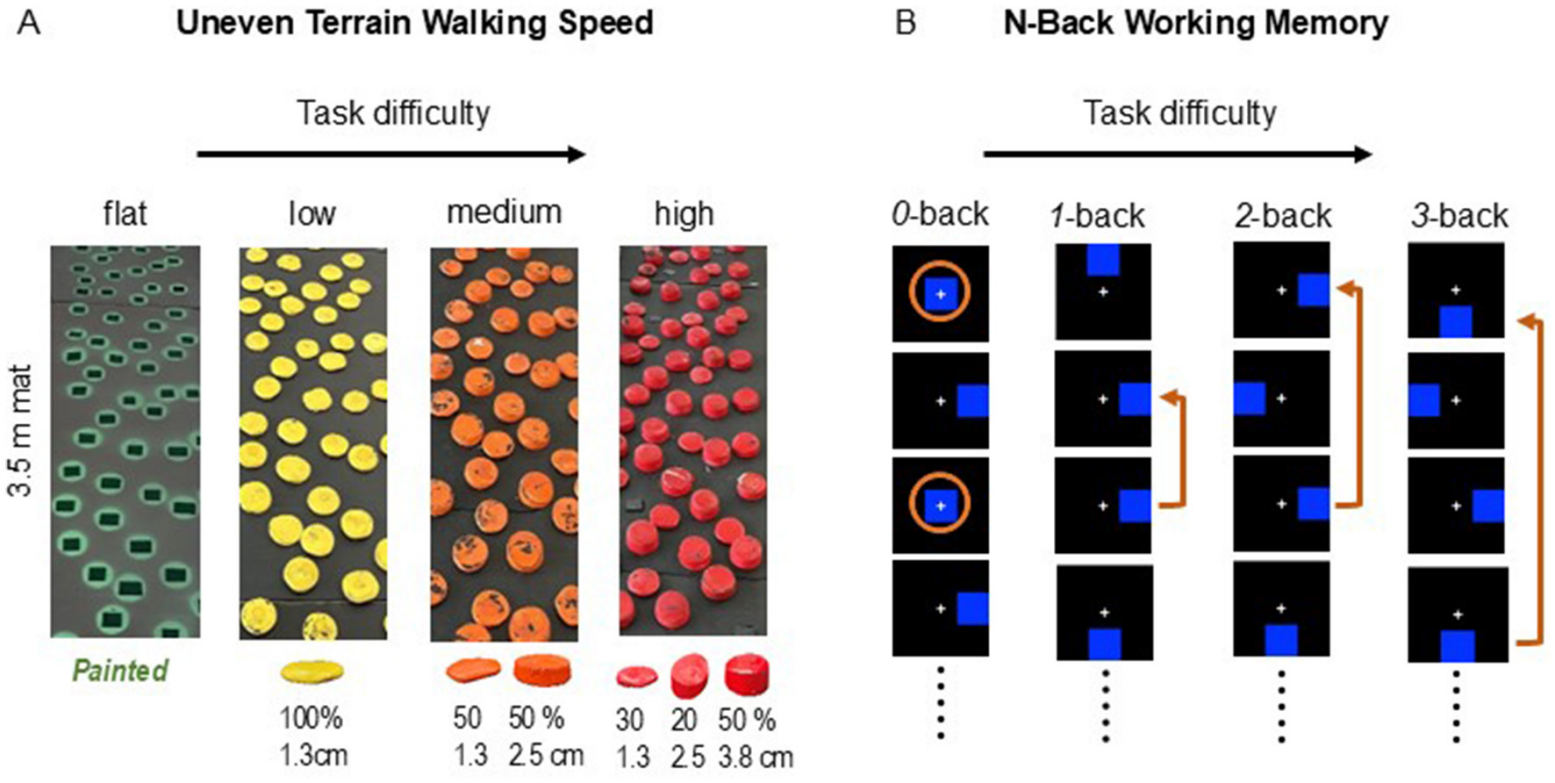
Schematic overview of the overground uneven terrain walking task **(A)** and the n-back task **(B)**. Participants walked at their typical pace over a novel custom-made overground uneven surface comprising four terrains: flat, low, medium, and high terrain unevenness while walking speed was measured. The four terrain surfaces were created by using rigid foam disks of various heights. The percentages indicate the proportion of disks of the specified height **(A)**. Participants performed the n-back task, including four n-back levels: 0-back, 1-back, 2-back, and 3-back, while prefrontal cortical activity was recorded with fNIRS **(B)**.

Four walking surface conditions (flat, low, medium, and high) were designed, each presenting distinct levels of uneven terrain (Clark et al., 2019; Downey et al., 2022). Uneven terrain was created using rigid foam disks of various heights (non-compressible, each 12.7 cm in diameter; Blockwire Manufacturing LLC, Goshen, AL, USA), which were attached to a 3.5 meter mat. The low uneven terrain consisted entirely of 1.3 cm tall disks painted in yellow. The medium uneven terrain consisted of 50% 1.3-cm tall and 50% 2.5-cm tall disks painted in orange. The high uneven terrain included three different height disks painted in red: 50% at 3.8 cm, 20% at 2.5 cm, and 30% at 1.3 cm. For the flat terrain, there were no disks on the walking surface, but green circles were painted on the mat to ensure that the visual aspect of the flat condition was similar to the other terrain conditions. Participants completed multiple practice trials for each terrain condition before testing and were then instructed to walk at a natural, comfortable pace over each terrain condition three times, with the order of conditions randomized. The time to complete the middle 3-m portion of the mat was measured with a stopwatch (Figure 1A).

### 2.5 Evaluation of cognitive function using the n-back task

To assess visuospatial working memory performance, we employed the spatial n-back task as shown in Figure 1B. Participants were seated comfortably in front of a computer monitor. During the n-back task, a blue square appeared on a computer screen in one of nine possible locations. The location of the square was refreshed every 500 ms for short inter-stimulus interval trials (“short ISI”) or 1,500 ms for long inter-stimulus interval trials (“long ISI”). Each time the square re-appeared, participants were instructed to indicate whether the current location matched the location presented n-back by pressing a designated key. For each n-back test, the location of the square was refreshed 16 times. For the 0-back test, participants were instructed to press the key only when the square appeared in the center position. For the 1-back test, participants were instructed to press the key only when the square appeared in the same position on two consecutive appearances. For the 2-back test, participants were instructed to press the key only when the square appeared in the same position as two appearances prior (regardless of the position at the most recent appearance). Likewise, the 3-back test required the participants to press the key when the square appeared in the same position as three appearances prior (regardless of the position at the two most recent appearances). If the positions did not match, participants were instructed to refrain from responding. Participants completed two runs of the n-back task. In the first run, the sequence was as follows: short ISI 0-back, long ISI 2-back, short ISI 3-back, long ISI 1-back, short ISI 1-back, short ISI 2-back, long ISI 0-back, and long ISI 3-back. Each n-back test was separated by a short rest period. The second run followed this sequence: short ISI 3-back, short ISI 1-back, long ISI 0-back, short ISI 2-back, short ISI 0-back, long ISI 2-back, long ISI 1-back, and long ISI 3-back. Each run lasted approximately 10 min, resulting in a total duration of approximately 20 min for the entire task.

For data analysis, we averaged one task from the first run and the corresponding task from the second run for each n-back level. We used custom R scripts (available at https://github.com/tfettrow/Crunch_Nback_Analysis) specifically to read and analyze *n*-back task data. The primary outcome, d-prime (*d*′), provides a net summary score of n-back performance by accounting for both successes and failures. The formula used for calculating *d*′ = Z_H_ – Z_FA_ (Macmillan and Creelman, 1990), where H and FA represent the “Hit” (correct) and “False Alarm” (false positive) rates, and Z signifies the Z-transformation. Hit rates denote the proportion of hits when a signal is present (hits/(hits + misses)), and False Alarm rates represent the proportion of false alarms when a signal is absent (false alarms/(false alarms + correct negative)) (Haatveit et al., 2010). When participants excel at maximizing hits (minimizing misses) and minimizing false alarms (maximizing correct rejections), their sensitivity in distinguishing between target and non-target stimuli during the task increases (Haatveit et al., 2010). Therefore, a high d-prime score indicates greater discriminability in signal/target detection, reflecting better overall performance on the n-back task (Figure 1B). We calculated d*-*prime with the R package Psycho (https://cran.r-project.org/web/packages/psycho/psycho.pdf). Additionally, reaction time (measured in ms) was recorded only for correct responses, reflecting the time participants took to respond to each stimulus during the task.

### 2.6 Assessment of prefrontal cortical activity using fNIRS

#### 2.6.1 fNIRS setup

Participants were outfitted with a commercially available multichannel continuous-wave fNIRS unit (OctaMon, Artinis Medical Systems, Nijmegen, Netherlands) to measure prefrontal cortical activity during the n-back task. The headband contained light sources emitting near-infrared light at continuous wavelengths of 760 and 850 nm, along with two near-infrared light detectors. Separate recording channels were distinguished by time-division multiplexing. The headband was positioned just above the eyebrows, with its midline aligned with the midline of the face. The source-detector optode location on the headband was fixed at 3.5 cm. Anatomical recording sites for each channel were estimated by measuring the mid-point location between each light emitter-detector pair, reported in reference to the International 10-10 system (Koessler et al., 2009). Horizontal placement in the transverse plane was measured as a percentage of head circumference, and vertical placement in the sagittal plane as a percentage of the nasion to inion distance. The group mean recording sites relative to the nasion were as follows for the vertical and horizontal directions, respectively: 21.4% ± 3.3 and 13.5% ± 2.5 (for the upper and lower optodes, respectively); 4.7% ± 0.5 and 9.3% ± 2.1 (for the inner and outer optodes, respectively). The medial lower left and right fNIRS optodes were approximately aligned with the landmarks of Fp1 and Fp2. The lateral lower left and right optodes were approximately aligned with the landmarks of AF7 and AF8. These measurement locations correspond to the medial and lateral subregions of Brodmann Area 10 (Koessler et al., 2009). The upper left and right fNIRS optodes (both medial and lateral) were approximately aligned with the landmarks of AF3 and AF4. These measurement locations correspond to Brodmann Area 9 (Koessler et al., 2009).

#### 2.6.2 fNIRS data acquisition

We recorded fNIRS data during the n-back task. Employing a block design, we alternated eight active blocks of testing with eight reference blocks of resting. Participants performed the n-back task during the active blocks, while they remained still and silent during the reference blocks.

Participants completed a total of 32 pairs of reference/active blocks across two run conditions, with 16 pairs completed for each run condition. Start and end points of each block were manually marked using a wireless remote device (PortaSync, Artinis Medical Systems, Nijmegen, Netherlands), which placed event markers in a separate recording channel that was time-synchronized to the fNIRS signals. The data were sampled at 10 Hz and exported to a computer for analysis.

#### 2.6.3 fNIRS data processing

We utilized a differential pathlength factor value of 6 in our fNIRS data analysis. Prefrontal oxyhemoglobin (O_2_Hb) concentrations were computed following the modified Beer-Lambert Law and analyzed using custom MATLAB programs (version R2015a, MathWorks, Natick, MA, USA). Raw fNIRS signals underwent preprocessing, including detrending and application of a low-pass filter with a cutoff frequency at 0.14 Hz to minimize physiological noise (Holtzer et al., 2011). A wavelet filter was employed to mitigate the impact of motion (Herold et al., 2018). Subsequently, a trained team member visually inspected the data, excluding any channels with evident signal quality issues (e.g., high amplitude artifacts inconsistent with physiological activity or no apparent change in signal). O_2_Hb concentration was used as the primary outcome due to its reliability and sensitivity compared with deoxyhemoglobin values (Miyai et al., 2001). Task-related changes in prefrontal cortical O_2_Hb (ΔO_2_Hb) were calculated for each participant and task. This process entailed averaging the two blocks of active O_2_Hb (during testing) and two blocks of resting O_2_Hb corresponding to each n-back level separately for the long ISI 1,500-ms task and the short ISI 500-ms n-back task, followed by computing the task-related change using the formula: ΔO_2_Hb = active O_2_Hb – resting O_2_Hb. The ΔO_2_Hb data from all channels were averaged for each participant and each task before subsequent analyses.

### 2.7 Statistical analysis

Baseline characteristics, including age, sex, MoCA, and 400-m assessments, underwent one-way analysis of variance for continuous data and chi-square (χ^2^) tests for categorical data. To investigate the impact of aging and mobility function on walking speed and *n*-back performance, a linear mixed model with subject-specific random intercepts was used to analyze the effects of group (younger adults, older adults with high physical function, and older adults with low physical function) as a between-subject factor, terrain condition (flat, low, medium, and high) or n-back level (0-back, 1-back, 2-back, and 3-back) as a repeated within-subject factor, and the interaction between group and terrain or n-back level on walking speed (m/s) and working memory performance (d-prime score and reaction time), respectively. Additionally, a linear mixed model was utilized to examine prefrontal ΔO_2_Hb (μM) as a dependent variable across n-back levels among groups. Before conducting statistical inference, we examined linearity through Q-Q plots and histograms and confirmed the normality of residual data using the Shapiro-Wilk test. If the group by terrain interaction achieved statistical significance, pairwise *post hoc* tests were conducted between groups for each terrain separately or between terrains for each group separately. However, in the absence of a significant interaction, the main effects of terrain and group were assessed independently for all combinations of terrains and groups. Pairwise comparisons were adjusted using the false discovery rate (FDR) correction method. Partial Eta squared ($\eta_{\text{p}}^{2}$) was obtained for effect size, with the following criteria applied: $\eta_{\text{p}}^{2}$ ≥ 0.01, $\eta_{\text{p}}^{2}$ ≥ 0.06, and $\eta_{\text{p}}^{2}$ ≥ 0.14 represent small, medium, and large effect sizes, respectively (Lakens, 2013).

A multivariate correlation analysis was conducted to assess the relationship between the percentage change in d-prime or reaction time from 0-back to 3-back and the percentage change in walking speed from flat to high terrain within each group for each n-back task by ISI condition. N-back performance, including d-prime scores and reaction time, as well as walking speed, was converted into percentage changes (%) to account for differences in measurement scales normalize the data across participants, and allow comparison relative to a baseline (Kelly et al., 2010). This approach allows changes across modalities to be interpreted on a common scale, rather than using non-comparable raw scores. The correlation coefficient (*r*) was used to quantify the strength of the relationship between changes in n-back performance (d-prime and reaction time) and uneven terrain walking speed. *P*-values were adjusted using the FDR correction method within each group, with corrections applied separately for the two outcome measures in the short and long ISI n-back tasks. The following definitions for effect sizes were employed (Cohen, 1992): *r* ≥ 0.10, *r* ≥ 0.30, and *r* ≥ 0.50 represent small, medium, and large effect sizes, respectively. Additionally, to compare correlation coefficients between groups, Fisher's *r*-to-*z* transformation was applied (Silver and Dunlap, 1987). Correlation coefficients were first converted into Fisher's *z*-scores, and the difference between the transformed values was computed. The standard error of the difference was estimated based on the sample sizes of each group. The *z*-difference score was then calculated and used to determine statistical significance with a two-tailed *P*-value. N-back data from two younger adults, two older adults with high physical function, and one older adult with low physical function were excluded due to technical issues (e.g., missing data or negative values at one or more n-back levels). Accordingly, these five participants were also excluded from both the correlation and fNIRS analyses. All statistical analyses were conducted using JMP software (JMP^®^ 15.0, SAS Institute Inc., Cary, NC, USA). The significance level (α) was set at 0.05.

## 3 Results

### 3.1 Between-group comparisons

As shown in Table 1, SPPB scores were significantly higher in the high physical function group compared to the low physical function group (*P* < 0.001), as expected due to group assignment based on this score. Age did not differ between the two physical function groups, and MoCA scores did not differ across all three groups. Additionally, the low physical function group had a significantly higher BMI and took longer to complete the 400-meter walk test compared to the high physical function group (BMI: *P* = 0.015, 400-meter walk test: *P* = 0.049) and the younger group (BMI: *P* < 0.001, 400-meter walk test: *P* = 0.005). However, both BMI and 400-meter walk times in the low physical function group remained within the established inclusion criteria. Sex distribution did not differ significantly between groups (χ^2^ = 3.59, *P* = 0.166), and therefore, sex was not included as a covariate in subsequent analyses.

**Table 1:** Participants' characteristics.

**Variable**	**Younger adults (Y)**	**Older adults high physical function (HM)**	**Older adults low physical function (LM)**	**Pairwise comparison**		
				**Y vs. HM**	**Y vs. LM**	**HM vs. LM**
Participants, *n*	24	29	15			
Sex (M/F), n^*^	11/13	14/15	3/12			
Age, years	22.8 ± 3.3	73.5 ± 4.9	75.1 ± 6.8	< 0.001	< 0.001	0.587
BMI, kg/m^2^	23.4 ± 3.3	25.5 ± 3.7	29.4 ± 5.5	0.172	< 0.001	0.015
SPPB, score	12.0 ± 0.0	11.0 ± 0.8	8.2 ± 0.8	< 0.001	< 0.001	< 0.001
400-m walk, min	5.7 ± 0.8	6.1 ± 1.0	6.8 ± 1.1	0.467	0.005	0.049
MoCA, score	28.0 ± 1.6	27.1 ± 1.6	27.3 ± 1.6	0.115	0.387	0.917

Data are presented as mean ± standard deviation (s.d.), except for participants and sex, which are reported as counts.

BMI, body mass index; SPPB, short physical performance battery; MoCA, Montreal cognitive assessment.

^*^Chi-square (χ^2^) tests were conducted to compare the distribution of sex between groups.

### 3.2 Overground uneven terrain walking speed

**Figure 2:**
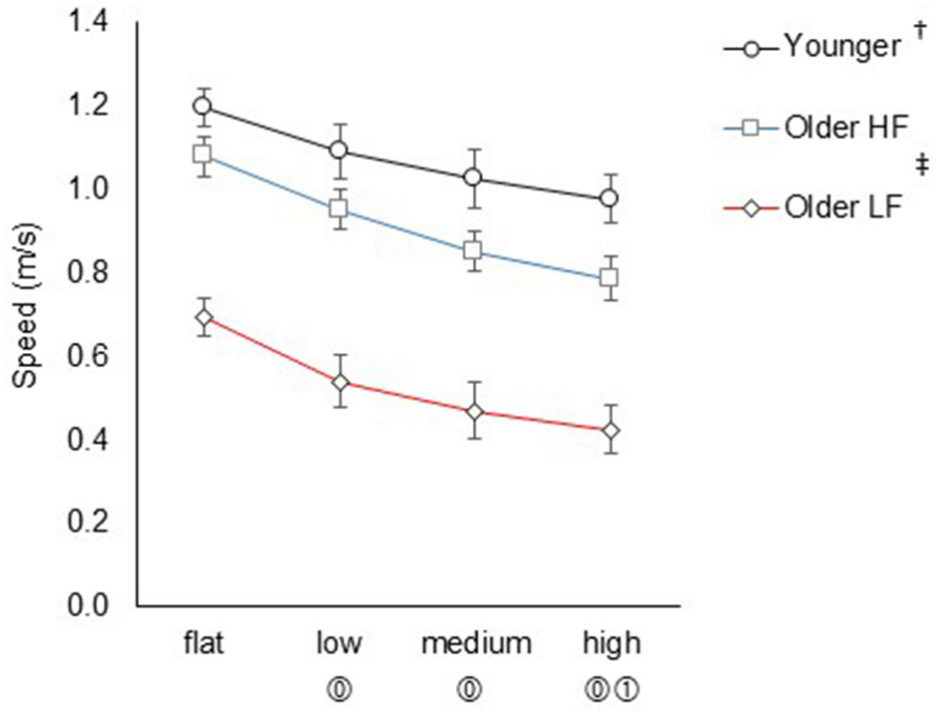
Overground uneven terrain walking speed across four terrain conditions. Data represent the mean ± standard error. The color of the lines represents each group. ^†^Indicates significantly different for the younger group compared to the older groups in all pooled terrains; ^‡^indicates significantly different between the older groups in all pooled terrains; ⓪ indicates significantly different compared to flat in all pooled groups; and ① indicates significantly different compared to low terrain in all pooled groups. HF refers to high physical function, and LF refers to low physical function.

A group × task interaction was not significant (*F*_6, 264_ = 0.11, *P* = 0.995, $\eta_{\text{p}}^{2}$ = 0.01), indicating that the effect of terrain unevenness on walking speed was similar across all three groups. The main effect of terrain (with data from all pooled groups) yielded significant results (*F*_3, 264_ = 10.58, *P* < 0.001, $\eta_{\text{p}}^{2}$ = 0.11), indicating a significant decrease in walking speed for low (_*FDRadj*._ *P* = 0.014), medium (_*FDRadj*._ *P* < 0.001), and high (_*FDRadj*._ *P* < 0.001) terrains compared with flat terrain, as well as for high terrain (_*FDRadj*._ *P* = 0.015) compared to low terrain. Additionally, the main effect of group (with data from all pooled terrains) yielded significant results (*F*_2, 264_ = 56.37, *P* < 0.001, $\eta_{\text{p}}^{2}$ = 0.31), indicating that overall walking speed was significantly slower in the low physical function group (_*FDRadj*._ *P* < 0.001) and the high physical function group (_*FDRadj*._ *P* < 0.001) compared to the younger group. Additionally, the low physical function group exhibited significantly slower walking speed compared to the high physical function group (_*FDRadj*._ *P* < 0.001; Figure 2). Descriptive data for terrain walking speed are available in the Supplementary Table S1.

### 3.3 N-back performance

#### 3.3.1 Long ISI (1,500 ms) n-back d-prime

**Figure 3:**
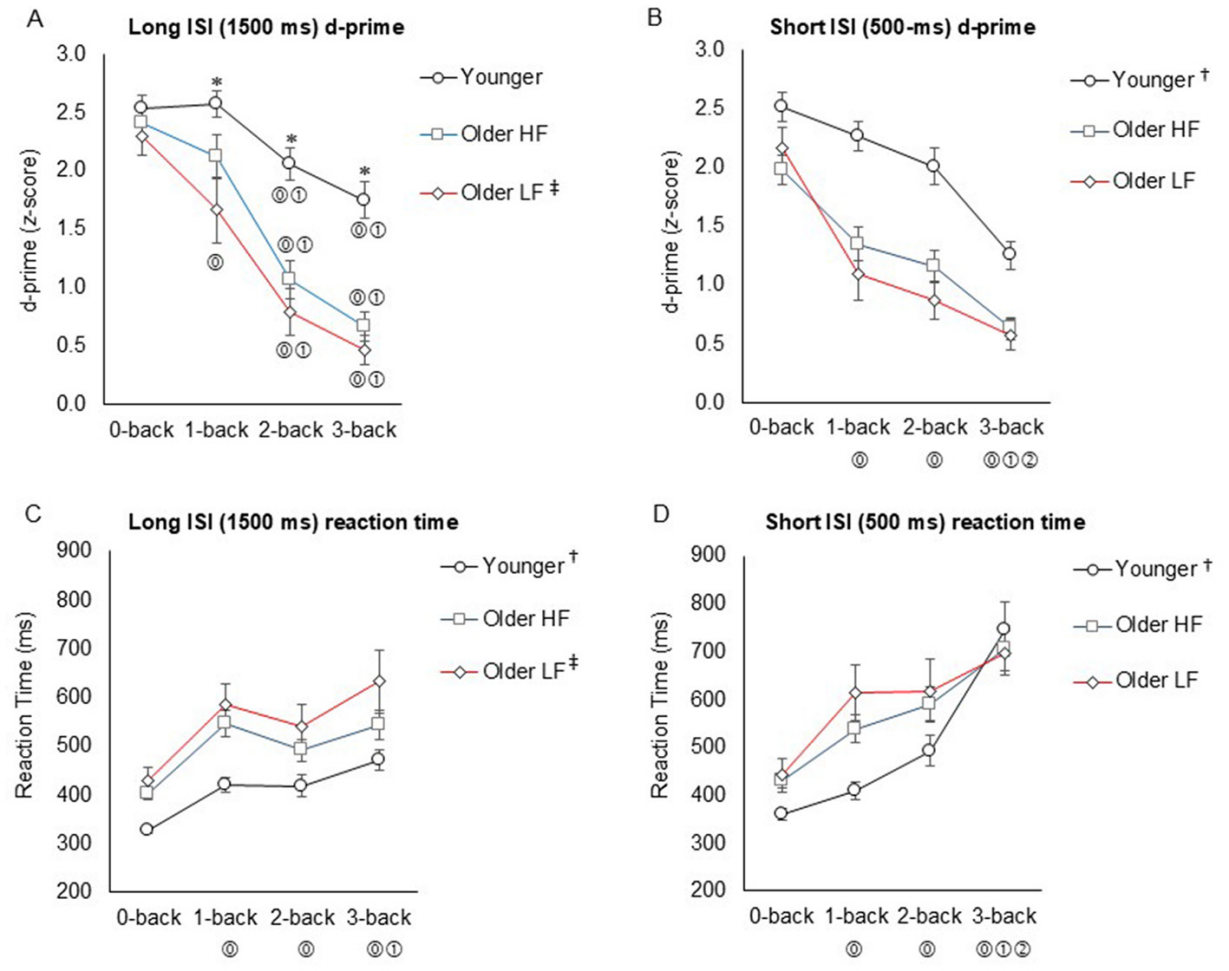
Change in d-prime scores (**A**: long ISI; **B**: short ISI) and reaction times (**C**: long ISI; **D**: short ISI) across n-back levels between groups. Data represent the mean ± standard error. ISI denotes inter-stimulus interval. Line colors represent each group. ^*^Indicates significantly greater d-prime scores for the younger group compared to the older groups at each n-back level. ⓪ Indicates significance compared to 0-back, ① indicates significance compared to 1-back, and ② indicates significance compared to 2-back within each group (if shown within the graph) or across all pooled groups (if located at the bottom of the graph). ^†^Indicates significantly different for the younger group compared to the older groups in all pooled n-back levels; ^‡^indicates significantly different between the older groups in all pooled n-back levels. HF refers to high physical function, and LF refers to low physical function.

A significant group × level interaction effect was observed for d-prime scores (*F*_6, 247_ = 3.14, *P* = 0.006, $\eta_{\text{p}}^{2}$ = 0.06), indicating that task difficulty affected groups differently, with lower d-prime scores reflecting reduced overall working memory performance. In the young group, d-prime scores decreased significantly at 2-back (_*FDRadj*._ *P* = 0.044) and 3-back (_*FDRadj*._ *P* < 0.001) compared to 0-back, and also at 2-back (_*FDRadj*._ *P* = 0.028) and 3-back (_*FDRadj*._ *P* < 0.001) compared to 1-back. The high physical function group exhibited similar decreases at 2-back (_*FDRadj*._ *P* < 0.001) and 3-back (_*FDRadj*._ *P* < 0.001) vs. 0-back, and also for 2-back (_*FDRadj*._ *P* < 0.001) and 3-back (_*FDRadj*._ *P* < 0.001) vs. 1-back. The low physical function group exhibited decreases starting from 1-back (_*FDRadj*._ *P* = 0.038) and continuing toward 2-back (_*FDRadj*._ *P* < 0.001) and 3-back (_*FDRadj*._ *P* < 0.001) vs. 0-back, and at 2-back (_*FDRadj*._ *P* = 0.004) and 3-back (_*FDRadj*._ *P* < 0.001) vs. 1-back. Between-group comparisons indicated larger decreases in both the low- and the high- physical function groups relative to the younger group across 1-back (_*FDRadj*._ *P* = 0.040 and *P* = 0.001, respectively), 2-back (_*FDRadj*._ *P* < 0.001 and *P* < 0.001, respectively), and 3-back (_*FDRadj*._ *P* < 0.001 and *P* < 0.001, respectively), with no significant difference between the older groups at any n-back level. However, the main effect of group (*F*_2, 247_ = 32.99, *P* < 0.001, $\eta_{\text{p}}^{2}$ = 0.22) showed that, across all pooled n-back levels, the low-functioning older group had lower d-prime scores than the high-functioning older group (_*FDRadj*._ *P* = 0.047; Figure 3A).

#### 3.3.2 Short ISI (500 ms) n-back d-prime

There was no significant group × n-back level interaction (*F*_6, 247_ = 1.54, *P*-= 0.166, = 0.04), indicating a similar effect of task difficulty across groups. The main effect of n-back level was significant (*F*_3, 247_ = 44.51, *P* < 0.001, $\eta_{\text{p}}^{2}$ = 0.36), with d-prime scores decreasing at 1-back (_*FDRadj*._ *P* < 0.001), 2-back (_*FDRadj*._ *P* < 0.001), and 3-back (_*FDRadj*._ *P* < 0.001) vs. 0-back, and 3-back vs. 1-back (*P* < 0.001) and 2-back (*P* < 0.001), pooled across groups. Additionally, the main effect of group (*F*_2, 247_ = 40.13, *P* < 0.001, $\eta_{\text{p}}^{2}$ = 0.25) showed lower d-prime scores in both the low physical function group (_*FDRadj*._ *P* < 0.001) and the high physical function group (_*FDRadj*._ *P* < 0.001) compared to the younger group, pooled across n-back levels, with no significant difference between the older groups (_*FDRadj*._ *P* = 0.424; Figure 3B).

#### 3.3.3 Long ISI (1,500 ms) n-back reaction time

The group × n-back level interaction was not significant (*F*_6, 247_ = 0.49, *P* = 0.815, $\eta_{\text{p}}^{2}$ = 0.01), indicating that task difficulty affected reaction times similarly across groups, with longer reaction times reflecting greater cognitive effort and slower processing. A significant main effect of n-back level was observed (*F*_3, 247_ = 19.19, *P* < 0.001, = 0.20), with reaction times increasing at 1-back (_*FDRadj*._ *P* < 0.001), 2-back (_*FDRadj*._ *P* < 0.001) and 3-back (_*FDRadj*._ *P* < 0.001) vs. 0-back, and at 3-back vs. 1-back (_*FDRadj*._ *P* = 0.014), pooled across groups. The main effect of group was significant (*F*_2, 248_ = 24.17, *P* < 0.001, $\eta_{\text{p}}^{2}$ = 0.17), showing longer overall reaction times in both the high physical function group (_*FDRadj*._ *P* < 0.001) and the low physical function group (_*FDRadj*._ *P* < 0.001) compared to the younger group. Additionally, the low physical function group exhibited longer overall reaction times than the high physical function group across all n-back levels (_*FDRadj*._ *P* = 0.031; Figure 3C).

#### 3.3.4 Short ISI (500 ms) n-back reaction time

The group × n-back level interaction was not significant (F_6, 247_ = 1.88, *P* = 0.085, = 0.05), indicating similar effects of task difficulty across groups. A significant main effect of n-back level was observed (*F*_3, 247_ = 30.63, *P* < 0.001, = 0.28), with longer reaction times at 1-back (_*FDRadj*._ *P* = 0.002), 2-back (_*FDRadj*._ *P* < 0.001), and 3-back (_*FDRadj*._ *P* < 0.001) compared to 0-back, and also at 3-back vs. 1-back (_*FDRadj*._ *P* < 0.001) and 2-back (_*FDRadj*._ *P* < 0.001), pooled across groups. The main effect of the group was also significant (*F*_2, 247_ = 5.34, *P* = 0.008, $\eta_{\text{p}}^{2}$ = 0.04), with both older groups showing longer overall reaction times than the younger groups (low functioning: _*FDRadj*._ *P* = 0.007; high functioning: _*FDRadj*._ *P* = 0.021). No significant difference was observed between the two older groups (_*FDRadj*._ *P* = 0.428; Figure 3D). Descriptive data for n-back performance are available in Supplementary Table S1.

### 3.4 The correlation between n-back performance and uneven terrain walking speed

**Figure 4:**
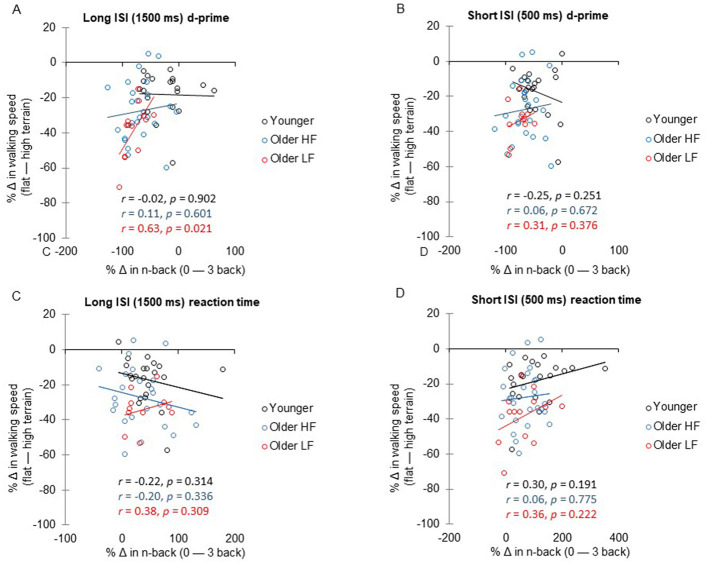
Multivariate correlation analysis illustrating the relationship between the percentage change (%Δ) in d-prime or reaction time from 0-back to 3-back and the percentage change in walking speed from flat to high terrain within each group. ISI denotes inter-stimulus interval. The text colors (black: younger group, blue: high physical function group, red: low physical function group) distinguish each group. The correlation coefficients (*r*) and *P*-values within each graph indicate the strength and significance of the relationship. HF refers to high physical function, and LF refers to low physical function.

For the long ISI (1,500 ms) n-back task, the low physical function group showed a significant positive correlation between the percentage change in d-prime from 0-back to 3-back and the percentage change in uneven terrain walking speed from flat to high terrain (*r* = 0.63, *P* = 0.021; Figure 4A), indicating a large effect size. This association remained significant after FDR correction (adjusted *P* = 0.032). No significant correlations were observed in the high physical function group (*r* = 0.11, *P* = 0.601) and the younger group (*r* = −0.02, *P* = 0.902). For the short ISI (500 ms) n-back task, the percentage change in d-prime was not significantly correlated with the percentage change in walking speed in any group (all *P* > 0.05; Figure 4B). Similarly, no significant associations were found between percentage changes in reaction time and walking speed for either ISI condition in any group (all *P* > 0.05; Figures 4C, D).

To compare correlation strengths across groups, Fisher's *r*-to-*z* transformation was applied for the significant long ISI correlation. The low physical function group showed a trend toward a stronger correlation compared to the high physical function group (*z* = 1.81, *P* = 0.070) and a significant stronger correlation compared to the younger group (*z* = 2.13, *P* = 0.033).

### 3.5 N-back task-related prefrontal cortical activity

#### 3.5.1 Prefrontal cortical activity during long ISI (1,500 ms) n-back task

**Figure 5:**
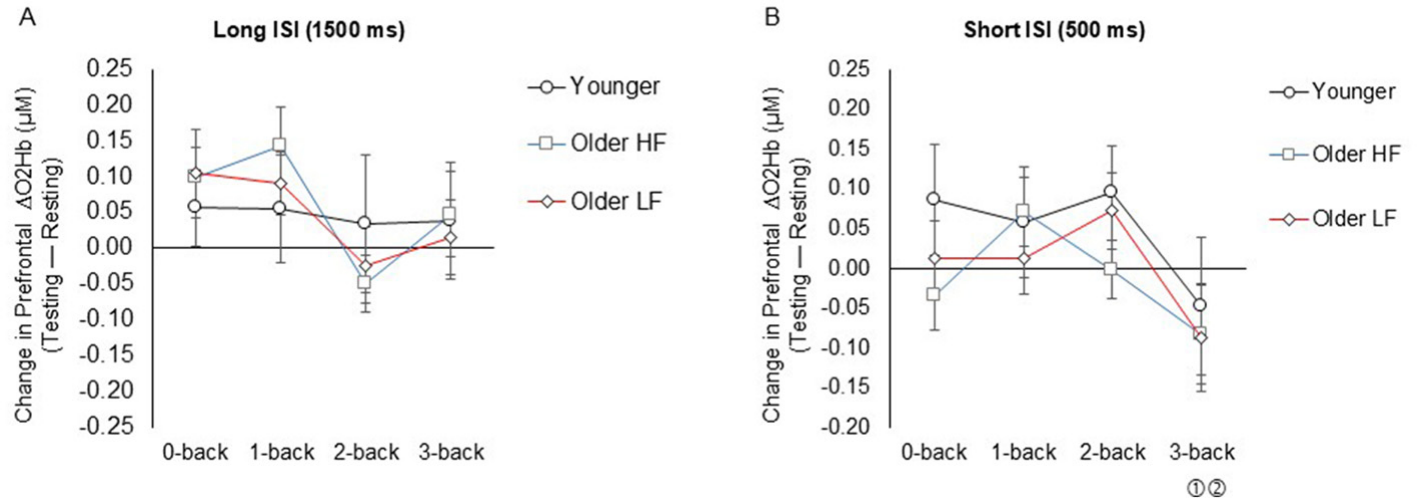
Task-related prefrontal cortical activity in long ISI 1,500-ms **(A)** and short ISI 500-ms **(B)** n-back tasks. Data represents the mean ± Standard error. ISI denotes inter-stimulus interval. ① Indicates significance compared to 1-back, and ② indicates significance compared to 2-back across all pooled groups. HF refers to high physical function, and LF refers to low physical function.

For the long ISI n-back task, the group × n-back level interaction was not significant (*F*_6, 243_ = 0.41, *P* = 0.870, $\eta_{\text{p}}^{2}$ = 0.01), indicating that task difficulty did not differentially affect prefrontal ΔO_2_Hb changes across groups. There were no significant main effects of n-back level (*F*_3, 243_ = 1.75, *P* = 0.158, $\eta_{\text{p}}^{2}$ = 0.02) or group (*F*_2, 243_ = 0.07, *P* = 0.935, $\eta_{\text{p}}^{2}$ = 0.01; Figure 5A).

#### 3.5.2 Prefrontal cortical activity during short ISI (500 ms) n-back task

Similarly, for the short ISI n-back task, the group × n-back level interaction was not significant (*F*_6, 243_ = 0.39, *P* = 0.884, $\eta_{\text{p}}^{2}$ = 0.01). While the main effect of group was not significant (*F*_2, 243_ = 1.22, *P* = 0.298, $\eta_{\text{p}}^{2}$ = 0.01), there was a significant main effect of n-back level (*F*_3, 243_ = 2.70, *P* = 0.047, $\eta_{\text{p}}^{2}$ = 0.03), with ΔO_2_Hb concentrations decreasing at 3-back compared to 1-back (_*FDRadj*._ *P* = 0.046) and 2-back (_*FDRadj*._ *P* = 0.046), pooled across all groups (Figure 5B). These null findings for both the long and short ISI tasks suggest that prefrontal cortical activation (ΔO_2_Hb) did not significantly differ by task difficulty across groups. Descriptive data for prefrontal cortical activity are available in Supplementary Table S1.

## 4 Discussion

The study results show that older adults exhibited slower uneven terrain walking speeds and poorer working memory performance compared to younger adults. These differences were particularly pronounced in older adults with low physical function. All groups exhibited slower walking speed as terrain became more uneven, and in older adults with low physical function, this slowing of walking speed was associated with poorer performance with increasing “*n*” on the n-back task. The effect sizes and statistical significance of this association were larger for the low physical function group, and not significant in the high physical function group or the younger group. Therefore, the major finding is an association between mobility and cognitive function that is specific to older adults, and particularly prominent in those with low physical function.

### 4.1 The impact of aging and mobility function on uneven terrain walking speed

As hypothesized, the present findings confirmed that walking speed gradually decreased as terrain became more uneven. This pronounced decline is likely attributable to a more cautious and attention-demanding gait pattern due to inconsistent biomechanical and sensory feedback when walking on an uneven surface (Downey et al., 2022; Shah et al., 2025). For example, participants likely choose to alter their typical foot placement locations relative to the discs, leading to step-to-step differences in step length and width, and different position of the foot and ankle joint (e.g., dorsi/plantar flexion and in/eversion). Collectively, these contribute to reduced walking speed (Downey et al., 2022; Hwang et al., 2024; Liu et al., 2024; Shah et al., 2025). We had expected a more rapid decline in walking speed in the low physical function group as terrain unevenness increased, but this was not observed, likely due to the group starting with significantly slower walking speed on flat terrain compared to the other groups.

### 4.2 The association between aging and mobility function with n-back working memory performance

N-back data showed a significant decline in d-prime scores—the primary performance measure—as task difficulty increased. This decline was notably more pronounced in the older groups compared to the younger group. In the short ISI n-back task, a significant main effect of the n-back level indicates that performance worsened as the n-back level increased across all pooled groups. This confirms that the task became more challenging as n-back level increased. Although there was a non-significant interaction effect, the overall decline in performance during the short ISI n-back task was greater in the older groups compared to the younger group across all pooled n-back levels, suggesting a detrimental effect of aging on the performance. This trend was more evident in the long ISI n-back task revealing a significant interaction effect. Younger adults exhibited a gradual decline in performance beginning at the 2-back level, whereas older adults, particularly those with low physical function, experienced a more rapid deterioration. Among older adults, those with low physical function showed performance declines beginning at the 1-back level, whereas those with high physical function began to decline at the 2-back level, progressing toward the most challenging 3-back level. The decline in d-prime scores indicates a diminished working memory capacity in terms of discriminability, particularly as task difficulty increases across all groups. The younger group exhibited a stronger ability to adapt to escalating cognitive demands, as evidenced by a slower decline in n-back performance compared to the older groups, who struggled more noticeably with increasing task difficulty. This finding highlights the increased difficulty of the task for older adults and underscores age-related deficits in working memory performance, linking age-related brain changes to alterations in memory and cognition (Nyberg et al., 2012). Additionally, a significant main effect of the group for the long ISI n-back task indicates that older adults with low physical function showed a substantially greater decline across all pooled n-back levels compared to their high physical function counterparts. This suggests that deficits in mobility function and cognitive function might be affected by the same underlying neurological impairments associated with aging.

Consistent with our findings, previous studies comparing age groups have reported a similar pattern. For instance, Gajewski et al. (2018) reported a significant interaction between age groups and n-back levels (0-back vs. 2-back) using a 1,500 ms inter-stimulus interval in the n-back task. Across the combined datasets, this study found that older participants (*n* = 194) had a higher proportion of missed targets at the 2-back level compared to younger participants (*n* = 157), while no significant group differences were observed at the 0-back level. Additionally, Hepdarcan and Can (2025) used a longer 2,000 ms inter-stimulus interval in the n-back task, which included 0-, 1-, 2-, and 3-back levels, and found that younger participants (*n* = 30) had better d-prime score than older participants (*n* = 25), although no significant interaction between age groups and n-back levels was found. However, Zajac-Lamparska et al. (2024) using a randomized inter-stimulus interval ranging from 1,800 to 2,500 ms inter-stimulus interval in the *n*-back task, which included 1-, 2-, and 3-back levels, found that both older participants (*n* = 50) and younger participants (*n* = 60) exhibited significantly lower d-prime scores at each difficulty level, including the easiest level (i.e., 1-back), following a similar pattern. Unlike previous studies comparing younger and older adults, our study's inclusion of older adults with low physical function may have contributed to the significant interaction between group and n-back level in the 1,500-ms n-back task. Study-specific differences in n-back task types should also be considered as a potential reason for inconsistent findings.

Additionally, n-back data revealed a significant increase in reaction time with increased n-back levels in both the long and short ISI conditions, with the effect particularly pronounced under the short ISI condition across all groups. This suggests that participants had greater difficulty responding to each stimulus as task demands increased, particularly when stimuli were refreshed every 500 ms in the short ISI condition compared to 1,500 ms in the long ISI condition. In the group comparison, the older groups consistently exhibited longer reaction times across all pooled n-back levels in both the long ISI (1,500 ms) and short ISI (500 ms) n-back tasks, compared to the younger group. Furthermore, significantly longer reaction times across all pooled n-back levels were observed in the low physical function group compared to the high physical function group during the long ISI n-back task. This observation aligns with previous studies showing that older adults typically exhibit slower reaction times in n-back task compared to younger individuals (Gajewski et al., 2018; Pergher et al., 2019; Hepdarcan and Can, 2025). This well-documented finding is attributed to age-related changes in brain function, specifically impacting processing speed in working memory, leading to a slower ability to identify and respond to target stimuli (Verhaeghen and Cerella, 2002). Essentially, the older brain takes longer to process information and make decisions (Eckert et al., 2010).

### 4.3 The relationship between uneven terrain walking speed and n-back working memory performance

As hypothesized, a novel finding in this study is the significant association, observed exclusively in the low physical function group, between slower uneven terrain walking speed and poorer n-back performance. This finding contrasts with the absence of a corresponding association in older adults with high physical function and younger adults. Particularly in the long ISI n-back task, a significant relationship was observed for older adults with a larger effect size evident in the low physical function group. Specifically, older adults with low physical function showed a significant association between the decline in walking speed from flat to high and the decline in n-back performance from 0-back 3-back. However, this relationship was not observed in older adults with high physical function or younger adults. An additional analysis examining whether the strength of the correlation differed between groups revealed that it was significantly stronger in the low physical function group compared to the younger group, and marginally stronger compared to the high physical function group. This finding suggests that declines in uneven terrain walking speed are more closely linked to declines in n-back working memory performance among older adults with low physical function. In the short ISI n-back task, declines in uneven terrain walking speed and in n-back performance were not significantly related in any of the groups. The long ISI condition likely imposes greater demands on working memory by requiring sustained rehearsal and increasing vulnerability to interference over a longer period of time (Vergauwe and Langerock, 2017). However, particularly in older groups, poorer performance in the short ISI condition suggests that the tasks may differ in factors beyond memory demands, such as perceptual or encoding challenges, or heightened attentional demands within a shorter timeframe (Buonomano et al., 2009). This may be due to the short ISI task being overly demanding, as shorter retention intervals can disrupt attentional refreshing in working memory, potentially leading to poorer performance (Camos et al., 2018), which could have weakened any observed relationship with uneven terrain walking speed.

The significant association in the long ISI n-back task performance with uneven terrain walking speed could be attributed to underlying neurophysiological impairments that affects both tasks, especially in the lower physical function group. Aging- or neurologically-related brain atrophy may play a role, as the motor and cognitive systems rely on shared neural resources, particularly in the prefrontal cortex and basal ganglia (Seidler et al., 2010). Neuroimaging studies employing cross-sectional and longitudinal approaches have shown age-related brain atrophy in cognitively normal individuals, characterized by a decline in volume and an accelerated atrophy rate across various brain structures (Scahill et al., 2003; Fujita et al., 2023). Notably, this neural loss is not equally distributed across the brain, with gray matter in the lateral prefrontal cortex being particularly susceptible to aging-related decline (Raz et al., 2004; Lemaitre et al., 2012). As aging or neurological conditions advance, the brain may prioritize either motor or cognitive tasks, leading to slower walking speed when cognitive demands increase, such as when navigating complex environments (Beurskens and Bock, 2012). One interpretation, therefore, is that brain impairments contribute to the decline in performance on both tasks. Furthermore, impairments in working memory, as assessed by the n-back task, may directly influence the use of working memory during uneven terrain walking. Walking on uneven terrain demands working memory for continuous updating, retention, attention, and adaptation, as individuals must constantly assess and adjust their movements to navigate complex environments such as uneven surfaces (Menant et al., 2014). This process involves simultaneously managing multiple pieces of information, such as the location of the discs on the uneven terrain walkway, the condition of the terrain, and the precise foot placement, to ensure stability while walking (Downey et al., 2022).

### 4.4 Prefrontal cortical activity during n-back working memory performance

The fNIRS results did not support the hypothesis that greater task demands in the n-back task would lead to a proportional increase in prefrontal cortical activity in either the short- or long-ISI condition nor did they reveal any significant group differences. Specifically, prefrontal cortical activity remained stable as n-back levels increased in the long ISI condition, while in the short ISI condition, activity was sustained through the 2-back level but declined significantly at the more demanding 3-back level across all pooled groups. The absence of a consistent increase in prefrontal cortical activity may suggest reduced task engagement or disengagement in response to increasing task difficulty, leading to lower cognitive effort as n-back levels increase. When task demands exceed participants' mental capacity, they may disengage and allocate fewer cognitive resources, resulting in lower brain activity—a downward trend that becomes more pronounced in more challenging tasks (Boere et al., 2024). This finding aligns with a previous fMRI study (Mattay et al., 2006) which demonstrated a similar distribution of prefrontal cortical activity between younger and older adults across all n-back task levels (1-, 2-, and 3-back levels), without a proportional increase in activation in either age group. Additionally, at higher task loads (2- and 3-back levels), older adults, who performed worse than younger adults in accuracy, exhibited relatively reduced prefrontal activity. Similarly, a recent fNIRS study using an n-back task with 1-, 2-, and 3-back levels (Zhu et al., 2024) found that older adults, who performed worse than younger adults only at the 2-back for accuracy, showed a trend of decreasing prefrontal cortical activity as task difficulty increased across the n-back levels; however, younger adults exhibited significant increases in prefrontal cortical activity at the 3-back relative to the 1-back. Consistent with our findings, older adults, in particular, showed no proportional increase in brain activation as working memory demands increased likely reflecting a decline in cognitive engagement and a corresponding reduction in cognitive effort in both long and short ISI conditions. The n-back task, particularly during the short ISI condition, may impose greater attentional demands, as reflected by longer mean reaction times compared to the 500 ms stimulus refresh interval, especially at higher n-back levels across all participant groups. This may reflect increased reliance on posterior brain regions due to temporal overlap (Eriksson et al., 2015), potentially limiting the interpretability of fNIRS measurements focused exclusively on the prefrontal cortex.

Additionally, the fNIRS results did not align with the hypothesis proposed by Reuter-Lorenz and Cappell (2008), which suggests that older adults engage in over-recruitment (greater brain activation than younger adults) at lower cognitive demands to sustain performance. Moreover, our findings do not support the notion that increasing task difficulty depletes neural resources in older adults, resulting in underactivation relative to younger adults. This is consistent with a recent electroencephalogram study (Zajac-Lamparska et al., 2024) analyzing theta and alpha wave power in the frontal-midline region during performance (d-prime) on an n-back task at three difficulty levels (1-, 2-, and 3-back levels), which did not support the hypothesis of compensatory brain activity in older adults compared to younger adults. The results indicated a reduced capacity to utilize neuronal resources relevant to the task in older adults, rather than showing compensatory activity. However, it is conceivable that age-related changes in brain areas involved in the n-back task vary across the adult lifespan. A meta-analysis of fMRI studies have shown that prefrontal cortex engagement during n-back task performance remains consistent in young adults, less so in middle-aged adults, and absent in older adults, suggesting a gradual decline in prefrontal cortex engagement with aging (Yaple et al., 2019). This could reflect a shift in resource allocation, with reduced reliance on the prefrontal cortex and increased involvement of other brain regions, such as the parietal cortex, dorsal cingulate cortex, insula, and cerebellum (Yaple et al., 2019), which may support compensatory activity in older adults (Cabeza et al., 2002; Reuter-Lorenz and Cappell, 2008). Furthermore, sustained engagement under higher cognitive demands may lead to mental fatigue, particularly in regions associated with cognitive effort, such as the prefrontal cortex, during tasks that require significant working memory and attentional resources (Yan et al., 2025). Therefore, the variability in prefrontal cortical recruitment in older adults may be influenced by a trade-off between task difficulty/modality, mental fatigue, and individual cognitive capacity.

### 4.5 Limitations

This cross-sectional study is limited in its ability to establish cause-and-effect relationships or track changes in variables over time. Consequently, longitudinal studies are needed to provide more comprehensive and definitive evidence regarding the relationship under investigation. Additionally, the relatively small sample size per group may reduce the reliability of the linear mixed model and correlation estimates and hinder the interpretation of hemodynamic responses measured by fNIRS during the n-back task, thereby limiting the generalizability of the findings. Other limitations include fNIRS's low spatial resolution and limited depth penetration, along with variability in probe placement across individuals, which affect detection of brain activity. Since we measured only prefrontal cortex activity, we cannot rule out contributions from other brain regions involved in working memory (Owen et al., 2005; Yaple et al., 2019). Furthermore, with regard to age-related compensatory mechanisms (Cabeza et al., 2018), our study design did not fully support detecting cognitive load-dependent differences in brain activity between older and younger adults. While cognitive performance is often related to brain activity, this relationship is not always straightforward and can be influenced by factors such as task modality, task complexity, individual differences, and external factors like stress, fatigue, and environmental conditions (Cabeza and Nyberg, 2000). Therefore, larger and more rigorously controlled studies are crucially required to validate age-related compensatory mechanisms.

## 5 Conclusions

The unique methodology employed in this study revealed an association between load-dependent declines of both uneven terrain walking speed and n-back performance, observed exclusively among older adults. This association was particularly pronounced in those with lower physical functional status, suggesting that although aging may be a primary factor, the link between mobility and cognitive function becomes more evident in lower-functioning older adults. These findings provide insight into shared neural mechanisms underlying age-related declines in mobility and cognitive function, informing the development of rehabilitation strategies to promote healthy aging.

## Data Availability

The raw data supporting the conclusions of this article will be made available by the authors, without undue reservation.
