# Supplementary material for: Associations between declines in uneven terrain walking speed and visuospatial working memory in older adults
Source: Front Aging Neurosci. 2026 Feb 2;17:1644741. doi: 10.3389/fnagi.2025.1644741 (PMC12907310; doi:10.3389/fnagi.2025.1644741)
Supplement: Supplementary file 1 [file Table_1.docx]

Supplementary Material

Supplemental Table 1: Descriptive Data

|  |  |  | Younger | | Older High Physical Function | | Older Low Physical Function | |
| --- | --- | --- | --- | --- | --- | --- | --- | --- |
| Variable | Condition | Task | Mean ± s.d. | | Mean ± s.d. | | Mean ± s.d. | |
| Walking Speed, m/s |  | flat | 1.2 | ±0.3 | 1.0 | ±0.3 | 0.8 | ±0.3 |
|  |  | low | 1.1 | ±0.3 | 0.9 | ±0.3 | 0.6 | ±0.3 |
|  |  | medium | 1.0 | ±0.3 | 0.8 | ±0.3 | 0.5 | ±0.3 |
|  |  | high | 1.0 | ±0.3 | 0.7 | ±0.3 | 0.5 | ±0.3 |
| d-prime, *z* | Long ISI (1500 ms) | 0-back | 2.5 | ±0.5 | 2.4 | ±0.6 | 2.3 | ±0.6 |
|  |  | 1-back | 2.6 | ±0.5 | 2.1 | ±1.0 | 1.7 | ±1.0 |
|  |  | 2-back | 2.1 | ±0.7 | 1.1 | ±0.8 | 0.8 | ±0.7 |
|  |  | 3-back | 1.7 | ±0.7 | 0.7 | ±0.7 | 0.5 | ±0.4 |
|  | Short ISI (500 ms) | 0-back | 2.5 | ±0.6 | 2.0 | ±0.7 | 2.2 | ±0.6 |
|  |  | 1-back | 2.3 | ±0.6 | 1.3 | ±0.7 | 1.1 | ±0.8 |
|  |  | 2-back | 2.0 | ±0.7 | 1.2 | ±0.7 | 0.9 | ±0.6 |
|  |  | 3-back | 1.3 | ±0.5 | 0.6 | ±0.5 | 0.6 | ±0.4 |
| Reaction Time, msec | Long ISI (1500 ms) | 0-back | 328.4 | ±38.8 | 403.8 | ±63.8 | 429.2 | ±102.8 |
|  |  | 1-back | 420.1 | ±68.7 | 543.0 | ±141.7 | 584.8 | ±146.4 |
|  |  | 2-back | 419.0 | ±101.9 | 500.7 | ±121.0 | 540.8 | ±160.2 |
|  |  | 3-back | 471.5 | ±92.5 | 543.0 | ±157.4 | 633.2 | ±215.6 |
|  | Short ISI (500 ms) | 0-back | 361.4 | ±54.8 | 430.2 | ±83.5 | 442.0 | ±123.9 |
|  |  | 1-back | 410.0 | ±84.4 | 538.4 | ±155.3 | 614.2 | ±209.5 |
|  |  | 2-back | 493.1 | ±145.1 | 590.7 | ±179.7 | 618.4 | ±226.1 |
|  |  | 3-back | 746.8 | ±260.9 | 707.1 | ±248.0 | 696.5 | ±168.5 |
| Prefrontal cortical activity, uM | Long ISI (1500 ms) | 0-back | 0.06 | ±0.2 | 0.10 | ±0.2 | 0.10 | ±0.2 |
|  |  | 1-back | 0.06 | ±0.3 | 0.14 | ±0.3 | 0.09 | ±0.2 |
|  |  | 2-back | 0.03 | ±0.4 | -0.05 | ±0.2 | -0.02 | ±0.2 |
|  |  | 3-back | 0.04 | ±0.4 | 0.05 | ±0.3 | 0.02 | ±0.2 |
|  | Short ISI (500 ms) | 0-back | 0.09 | ±0.3 | -0.03 | ±0.2 | 0.01 | ±0.2 |
|  |  | 1-back | 0.06 | ±0.3 | 0.07 | ±0.2 | 0.01 | ±0.2 |
|  |  | 2-back | 0.09 | ±0.3 | 0.00 | ±0.2 | 0.07 | ±0.2 |
|  |  | 3-back | -0.05 | ±0.4 | -0.08 | ±0.3 | -0.09 | ±0.2 |
